# Hemodynamic Effects of Ketamine Infusion in the Intensive Care Unit for Maintenance Sedation Compared With Propofol and Midazolam: A Retrospective Cohort Study

**DOI:** 10.31486/toj.22.0032

**Published:** 2022

**Authors:** Sohaib Khatib, David Roelofsz, Som Singh, Arjun Rao, Taylor Brinton, Gregory Howell

**Affiliations:** ^1^ Department of Internal Medicine, University of Missouri-Kansas City School of Medicine, Kansas City, MO; ^2^ University of Missouri-Kansas City School of Medicine, Kansas City, MO; ^3^ Department of Critical Care Medicine, Banner Thunderbird Medical Center, Glendale, AZ; ^4^ Department of Pulmonary and Critical Care Medicine, University of Missouri-Kansas City School of Medicine, Kansas City, MO

**Keywords:** *Hemodynamics*, *infusions–intravenous*, *intensive care units*, *ketamine*, *midazolam*, *propofol*

## Abstract

**Background:** Sedation and analgesia in the intensive care unit (ICU) are major clinical challenges, and several continuous infusion medications have been used for these purposes. The use of these sedative medications has been associated with hemodynamic effects that complicate the patient's critical illness. Continuous ketamine infusion is an emerging sedative option that has been used more frequently in the ICU since 2017. The purpose of this study was to characterize the hemodynamic differences between 3 continuous sedative infusions: ketamine, propofol, and midazolam.

**Methods:** For this single-center retrospective cohort study, we collected data for patients hospitalized between January 2015 and April 2020 at Saint Luke's Health System in Kansas City, Missouri. Adult patients in the ICU requiring a norepinephrine infusion and sedation were included. The change in norepinephrine requirement from baseline at 1 hour was the primary outcome. The change in vasopressor requirement at 3 and 30 hours after initiation of the infusion was also tabulated.

**Results:** Sixty-eight critically ill patients with several types of shock requiring vasopressor support with norepinephrine were enrolled in our study. Patients who received ketamine had an increase in norepinephrine requirement compared to midazolam and propofol, although this difference was not statistically significant.

**Conclusion:** In our study, continuous ketamine infusion did not reveal a statistically significant favorable hemodynamic effect compared with propofol and midazolam because of the small sample size. A trend toward an unfavorable hemodynamic effect is not expected, but large randomized trials are needed to further evaluate the hemodynamic effects of continuous ketamine infusion in the ICU.

## INTRODUCTION

Sedation is a staple of the intensive care unit (ICU) and has been for decades, with the intention of promoting the comfort and safety of critically ill patients. Today, several options for sedation are available, including benzodiazepines, opioid analgesics, dexmedetomidine, ketamine, and antipsychotics. The use of each is related both to the relative indication (ie, pain, delirium, agitation) and to the side effect profile of each agent that may limit its use in particular clinical scenarios.

Because of the relatively rapid onset and elimination of propofol, this drug is often the sedative of choice in the ICU setting. A large, randomized trial comparing lorazepam vs propofol with daily interruptions found that propofol decreased mean ventilator days,^1^ and a multicenter database analysis of more than 3,000 patients found that propofol infusions when compared with midazolam or lorazepam were associated with lower mortality, earlier discharge, and discontinuation from mechanical ventilation.^2^ With this mounting evidence of superiority, propofol is often the choice for sedation.

The use of propofol has limiting aspects, however. Propofol-related syndrome, a rare complication of propofol infusion, is associated with bradycardia, cardiovascular collapse, rhabdomyolysis, and renal failure.^3^ Hypotension, a side effect associated with propofol use, occurs in about 25% of cases^4^ and results in an increased requirement for vasopressors and prolonged duration of vasopressor use, thereby increasing the risk of morbidity associated with propofol use.

Ketamine, an emerging sedative with some analgesic properties, is not associated with hypotension as propofol is.^5^ Theoretically then, ketamine may be a better choice for patients with hemodynamic compromise. However, there is a paucity of information in the literature identifying when ketamine might be a better choice in clinical scenarios of hypotension for avoiding prolonged or high doses of vasopressors. We sought to define the role of ketamine in relation to relative vasopressor requirements after the initiation of sedation and to compare propofol, ketamine, and midazolam in their tendency to require higher doses of vasopressors for longer periods of time.

## METHODS

### Study Design and Setting

We performed this single-center retrospective cohort study of patients hospitalized from January 1, 2015, to April 29, 2020, at Saint Luke's Health System, which includes 3 hospitals in Kansas City, Missouri. We gathered information regarding the use of sedative medications in the ICU from the Saint Luke's Health System database.

### Study Population and Sample Size

In the period 2015 to 2020, ketamine infusion was used for only 22 patients who were in shock and required vasopressor support with norepinephrine. We included all these patients in the ketamine group. For comparison, we included 24 patients who required continuous sedation with propofol and 22 patients who received midazolam for sedation. We chose the patients in the propofol and midazolam groups using the random number method. Every patient was assigned a number, and using the random number function in Excel (Microsoft Corporation), we randomly picked the included patients from the pool of patients who met the inclusion criteria. We defined 3 inclusion criteria: patients had to (1) be critically ill with a confirmed diagnosis of shock requiring vasopressor support with only norepinephrine; (2) require continuous sedation with either ketamine, propofol, or midazolam; and (3) be ≥18 years of age. We excluded patients who required more than 1 vasopressor and those on more than 1 continuous sedative medication.

### Data Sources and Data Collection Procedure

After obtaining approval of our protocol from the Institutional Review Board of Saint Luke's Health System and permission from the education department of Saint Luke's Health System, we obtained access to patient medical records. We collected the following clinical features for the included patients: age, sex, shock type, and outcome (deceased vs discharged). For each patient included in the study, we collected the doses of epinephrine at 4 time points: baseline (time 0 before starting the continuous sedative infusion) and at 1, 3, and 30 hours after starting the continuous sedative medication. We used the changes in vasopressor doses as a measure of hemodynamic effect rather than conventional continuous blood pressure monitoring. Blood pressure does not always equate to blood flow, while vasopressor doses reflect the effective tissue perfusion and are therefore a more reliable reflection of hemodynamics.^6^

### Statistical Analysis

We present baseline patient characteristics of the ketamine, propofol, and midazolam groups. The median (interquartile range) doses of vasopressor at baseline and at 1, 3, and 30 hours after the initiation of the intervention are presented by treatment group. The differences in the least square means between the intervention groups and the corresponding 95% CI at each time point adjusted for the baseline doses of vasopressor, age, sex, and shock types were calculated to compare the hemodynamic effects between the groups. Analyses were performed with SAS software, version 9.4 (SAS Institute, Inc.). A *P* value <0.05 was considered statistically significant.

A sample size of 22 for each group was projected to achieve 80% power with α=0.05 to detect a difference in the mean dose of vasopressor between the 2 groups of at least 0.080, 0.088, and 0.15 units from baseline to 1, 3, and 30 hours, respectively. The assumptions for this calculation were derived from the estimated standard deviation for vasopressor doses among the 68 patients in the analyses.

## RESULTS

A total of 68 patients admitted to the Saint Luke's Health System between 2015 and 2020 with various types of shock requiring vasopressor support and sedation were included in this retrospective study: 22 patients in the ketamine group, 24 in the propofol group, and 22 in the midazolam group (Table 1). The mean age of patients in our study was 53.3 years. The ketamine group (63.6%) and propofol group (79.2%) had more male patients than females, but the midazolam group had an equal number of males and females. In the propofol group, the major shock type was septic shock (50%), whereas cardiogenic shock was the major type of shock in the midazolam group (40.9%). Overall, 27 patients (39.7%) did not survive the ICU admission. Patients in the ketamine group had the highest survival rate (77.3%), followed by 59.1% in the midazolam group and 45.8% in the propofol group.

**Table 1.** Patient Characteristics by Intervention Group

Variable	Ketamine Group, n=22	Propofol Group, n=24	Midazolam Group, n=22
Age, years, mean ± SD	47.9 ± 16.4	59.3 ± 9.0	52.8 ± 16.0
Sex			
Female	8 (36.4)	5 (20.8)	11 (50.0)
Male	14 (63.6)	19 (79.2)	11 (50.0)
Shock type			
Septic	10 (45.5)	12 (50.0)	7 (31.8)
Cardiogenic	1 (4.5)	6 (25.0)	9 (40.9)
Other (eg, obstructive, distributive)	11 (50.0)	6 (25.0)	6 (27.3)
Initial dose, mean ± SD	0.3 ± 0.3, mg/kg/h	27.3 ± 18.3, μg/kg/min	4.3 ± 4.2, mg/h
Outcome			
Deceased	5 (22.7)	13 (54.2)	9 (40.9)
Discharged	17 (77.3)	11 (45.8)	13 (59.1)

Note: Data are presented as n (%) unless otherwise indicated.

### Association of Sedative Medications With Vasopressor Requirements

The median norepinephrine requirement at baseline was lower in the ketamine group compared with the propofol and midazolam groups. Median norepinephrine doses continued to be lower in the ketamine group vs the propofol and midazolam groups at 1, 3, and 30 hours after starting the sedative medication, although the nor-epinephrine requirement increased from baseline in the ketamine group compared to the propofol and midazolam groups. Table 2 and the Figure show the doses of vasopressor over time after the initiation of different sedative medications.

**Table 2.** Norepinephrine Doses by Intervention Group

	Norepinephrine Dose, μg/kg/min		
Timepoint	Ketamine Group, n=22	Propofol Group, n=24	Midazolam Group, n=22
Baseline, 0 minutes	0.050 (0.010, 0.080)	0.100 (0.060, 0.325)	0.090 (0.040, 0.220)
1 hour	0.050 (0.040, 0.090)	0.100 (0.060, 0.210)	0.075 (0.050, 0.200)
3 hours	0.060 (0.020, 0.090)	0.080 (0.045, 0.200)	0.085 (0.040, 0.220)
30 hours	0.020 (0.000, 0.050)	0.075 (0.040, 0.100)	0.055 (0.010, 0.100)

Note: Data are reported as median (interquartile range).

**Figure.**
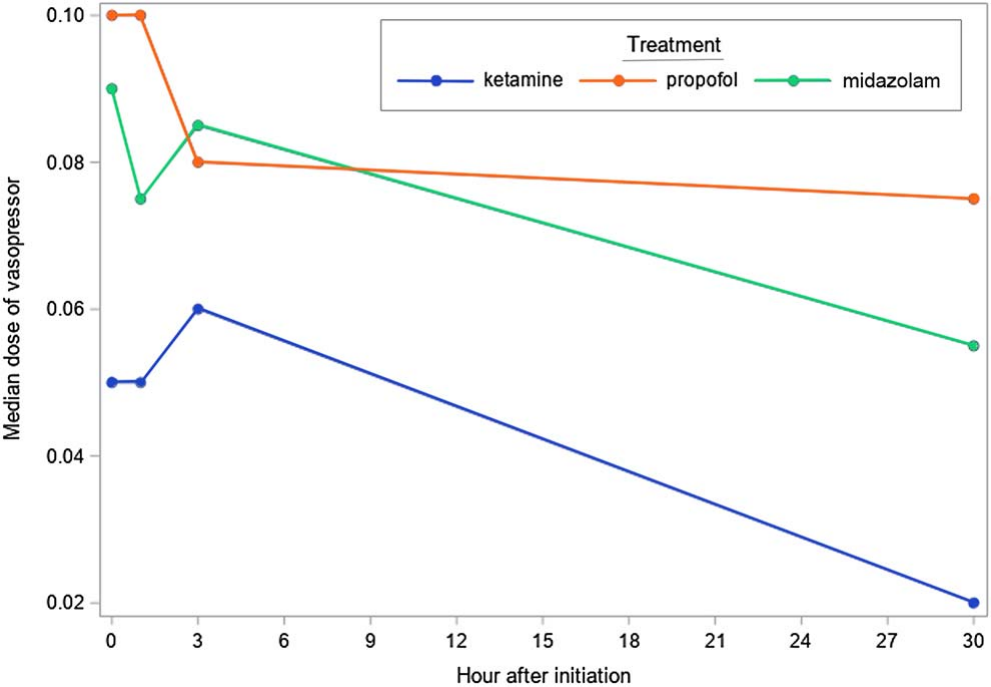
Line plot of the median doses of vasopressor over time after initiation by intervention group.

### Changes in Vasopressor Requirements Between Sedative Medications From Baseline

Table 3 shows the changes in norepinephrine doses from baseline at 3 time points in the ketamine vs propofol groups and the ketamine vs midazolam groups. We found no statistically significant differences in norepinephrine dose changes between groups.

**Table 3.** Changes From Initiation of Vasopressor Doses

Infusion Comparison/Time Points	Least Square Mean (SE)		Least Square Mean Difference	95% CI for Difference	*P* Value
Propofol vs ketamine	**Propofol**	**Ketamine**			
Baseline	0.164 (0.041)	0.050 (0.044)	0.114	(–0.005, 0.233)	0.061
Baseline to 1 h	–0.017 (0.023)	–0.023 (0.025)	0.006	(–0.061, 0.073)	0.863
Baseline to 3 h	–0.009 (0.023)	–0.009 (0.025)	–0.0003	(–0.067, 0.067)	0.992
Baseline to 30 h	–0.092 (0.023)	–0.073 (0.025)	–0.019	(–0.086, 0.048)	0.582
Midazolam vs ketamine	**Midazolam**	**Ketamine**			
Baseline	0.186 (0.039)	0.050 (0.044)	0.142	(0.015, 0.257)	0.028
Baseline to 1 h	–0.011 (0.023)	–0.023 (0.025)	0.012	(–0.057, 0.081)	0.732
Baseline to 3 h	–0.009 (0.023)	–0.009 (0.025)	0.00008	(–0.069, 0.069)	0.998
Baseline to 30 h	–0.070 (0.023)	–0.073 (0.025)	0.003	(–0.065. 0.072)	0.925

Note: Model adjusted for baseline dose of vasopressor, age, sex, and shock type.

## DISCUSSION

The hemodynamic effects of continuous ketamine infusion,^7-10^ continuous propofol infusion,^11-13^ and continuous midazolam infusion^14^ in critically ill patients have been evaluated in prior studies. To our knowledge, ours is the first study to compare the effects of these 3 continuous infusions on hemodynamics by examining the changes in vasopressor doses.

In our study, we included patients with different types of shock: septic, cardiogenic, and other (such as obstructive and distributive shock). We used the norepinephrine requirement as a reliable measure to reflect the hemodynamics of critically ill patients in shock.^15^ In our health system during the 5-year study period, ketamine infusion was used in 22 critically ill patients requiring vasopressor support with norepinephrine. The median requirement of norepinephrine in the ketamine group was lower at baseline and at 1, 3, and 30 hours vs the propofol and midazolam groups. These findings are concerning for selection bias among the ketamine group, as these patients seem to have had a lower severity of illness^16^ given their lower vasopressor doses. One explanation could be that intensivists tend to use other sedative medications such as propofol, midazolam, or dexmedetomidine infusions early when patients are acutely and critically ill and are usually on higher doses of vasopressors, while ketamine infusion is usually used later during the ICU stay when the acute critical issue has improved. This tendency could explain the lower doses of vasopressors and higher survival rate among the patients in the ketamine group. Interestingly, the norepinephrine requirement increased in the ketamine group and decreased in the propofol and midazolam groups. This finding is contradictory to what we expected, although the difference was not statistically significant.

Studies have found that continuous ketamine infusion has favorable hemodynamic effects, as it reduces vasopressor requirements^17^ and results in less clinically relevant hypotension compared to propofol.^18^ On the other hand, continuous propofol infusion can result in a negative hemodynamic event.^11^

Our work has several limitations. While our results did not reveal a statistically significant difference in the changes in norepinephrine doses from baseline among the 3 sedative medications, our findings are limited by the small sample size. Also, our results are concerning in terms of selection bias, especially in the ketamine group. The absence of control groups (eg, critically ill patients without shock or shock patients requiring vasopressor support with other medications) limits the interpretation of the hemodynamic effects of continuous sedative medications. Finally, some clinical factors might affect the hemodynamic effects of sedative medications that we did not examine in our study, such as the length of ICU stay, the use of other medications, and the role of other medical comorbidities.

## CONCLUSION

Continuous ketamine infusion has been used more frequently in the ICU since 2017 for sedation and has been described as having a favorable hemodynamic effect. We did not find a statistically significant difference in hemodynamic effects in our comparison of ketamine, propofol, and midazolam. In fact, we noted a trend toward increased vasopressor requirements in the ketamine group compared to the propofol and midazolam groups. Randomized clinical trials with large samples and more controlled settings are needed to evaluate the hemodynamic effects of continuous sedative medications in the ICU.

## Notes

*This article meets the Accreditation Council for Graduate Medical Education and the American Board of Medical Specialties Maintenance of Certification competencies for Patient Care and Medical Knowledge.*

## References

[1] Carson SS, Kress JP, Rodgers JE, A randomized trial of intermittent lorazepam versus propofol with daily interruption in mechanically ventilated patients. Crit Care Med. 2006;34(5):1326-1332. doi: 10.1097/01.CCM.0000215513.63207.7F 16540958

[2] Lonardo NW, Mone MC, Nirula R, Propofol is associated with favorable outcomes compared with benzodiazepines in ventilated intensive care unit patients [published correction appears in Am J Respir Crit Care Med. 2014 Jun 1;189(11):e70]. Am J Respir Crit Care Med. 2014;189(11):1383-1394. doi: 10.1164/rccm.201312-2291OC 24720509

[3] Chen L, Lim FA. Propofol infusion syndrome: a rare but lethal complication. Nursing. 2014;44(12):11-13. doi: 10.1097/01.NURSE.0000456376.94907.11 25406773

[4] Ozkoçak I, Altunkaya H, Ozer Y, Ayoğlu H, Demirel CB, Ciçek E. Comparison of ephedrine and ketamine in prevention of injection pain and hypotension due to propofol induction. Eur J Anaesthesiol. 2005;22(1):44-48. doi: 10.1017/s0265021505000104 15816573

[5] Ferguson I, Bell A, Treston G, New L, Ding M, Holdgate A. Propofol or ketofol for procedural sedation and analgesia in emergency medicine-the POKER study: a randomized double-blind clinical trial. Ann Emerg Med. 2016;68(5):574-582.e1. doi: 10.1016/j.annemergmed.2016.05.024 27460905

[6] Hollenberg SM. Vasopressor support in septic shock. Chest. 2007;132(5):1678-1687. doi: 10.1378/chest.07-0291 17998371

[7] Groetzinger LM, Rivosecchi RM, Bain W, Ketamine infusion for adjunct sedation in mechanically ventilated adults. Pharmacotherapy. 2018;38(2):181-188. doi: 10.1002/phar.2065 29193185

[8] Park S, Choi AY, Park E, Effects of continuous ketamine infusion on hemodynamics and mortality in critically ill children. PLoS One. 2019;14(10):e0224035. doi: 10.1371/journal.pone.0224035 31626685 PMC6799949

[9] Miller AC, Jamin CT, Elamin EM. Continuous intravenous infusion of ketamine for maintenance sedation. Minerva Anestesiol. 2011;77(8):812-820. 21730929

[10] Amer M, Maghrabi K, Bawazeer M, Adjunctive ketamine for sedation in critically ill mechanically ventilated patients: an active-controlled, pilot, feasibility clinical trial. J Intensive Care. 2021;9(1):54. doi: 10.1186/s40560-021-00569-1 34462007 PMC8404029

[11] Benken S, Madrzyk E, Chen D, Hemodynamic effects of propofol and dexmedetomidine in septic patients without shock. Ann Pharmacother. 2020;54(6):533-540. doi: 10.1177/1060028019895502 31849243

[12] Nelson KM, Patel GP, Hammond DA. Effects from continuous infusions of dexmedetomidine and propofol on hemodynamic stability in critically ill adult patients with septic shock. J Intensive Care Med. 2020;35(9):875-880. doi: 10.1177/0885066618802269 30260732

[13] Erdman MJ, Doepker BA, Gerlach AT, Phillips GS, Elijovich L, Jones GM. A comparison of severe hemodynamic disturbances between dexmedetomidine and propofol for sedation in neurocritical care patients. Crit Care Med. 2014;42(7):1696-1702. doi: 10.1097/CCM.0000000000000328 24717468

[14] Yao L, Liu B, He T, Shen F. Comparative study of dexmedetomidine vs. midazolam on plasma catecholamine levels and hemodynamics in patients with septic shock. Article in Chinese. Zhonghua Wei Zhong Bing Ji Jiu Yi Xue. 2021;33(10):1193-1197. doi: 10.3760/cma.j.cn121430-20210119-00105 34955127

[15] Benchekroune S, Karpati PC, Berton C, Diastolic arterial blood pressure: a reliable early predictor of survival in human septic shock. J Trauma. 2008;64(5):1188-1195. doi: 10.1097/TA.0b013e31811f3a45 18469640

[16] Garber PM, Droege CA, Carter KE, Harger NJ, Mueller EW. Continuous infusion ketamine for adjunctive analgosedation in mechanically ventilated, critically ill patients. Pharmacotherapy. 2019;39(3):288-296. doi: 10.1002/phar.2223 30746728

[17] Garner O, Patterson J, Mejia JM, Impact of ketamine as an adjunct sedative in acute respiratory distress syndrome due to COVID-19 pneumonia. Respir Med. 2021;189:106667. doi: 10.1016/j.rmed.2021.106667 34757277 PMC8552750

[18] Atchley E, Tesoro E, Meyer R, Bauer A, Pulver M, Benken S. Hemodynamic effects of ketamine compared with propofol or dexmedetomidine as continuous ICU sedation. Ann Pharmacother. 2022;56(7):764-772. doi: 10.1177/10600280211051028 34670425

